# Endothelin-1 Predicts Hemodynamically Assessed Pulmonary Arterial Hypertension in HIV Infection

**DOI:** 10.1371/journal.pone.0146355

**Published:** 2016-01-11

**Authors:** Rushi V. Parikh, Yifei Ma, Rebecca Scherzer, Amanda S. Heringer, John S. Macgregor, Jeffrey N. Martin, Steven G. Deeks, Peter Ganz, Priscilla Y. Hsue

**Affiliations:** 1 Department of Cardiovascular Medicine, Stanford University, Stanford, California, United States of America; 2 Department of Pediatrics, University of California San Francisco, San Francisco, California, United States of America; 3 Division of Endocrinology and Metabolism, San Francisco VA Medical Center, San Francisco, California, United States of America; 4 San Francisco General Hospital Cardiology Division, University of California San Francisco, San Francisco, California, United States of America; 5 Department of Epidemiology and Biostatistics, University of California San Francisco, San Francisco, California, United States of America; 6 San Francisco General Hospital Positive Health Program, University of California San Francisco, San Francisco, California, United States of America; Vanderbilt University Medical Center, UNITED STATES

## Abstract

**Background:**

HIV infection is an independent risk factor for PAH, but the underlying pathogenesis remains unclear. ET-1 is a robust vasoconstrictor and key mediator of pulmonary vascular homeostasis. Higher levels of ET-1 predict disease severity and mortality in other forms of PAH, and endothelin receptor antagonists are central to treatment, including in HIV-associated PAH. The direct relationship between ET-1 and PAH in HIV-infected individuals is not well described.

**Methods:**

We measured ET-1 and estimated pulmonary artery systolic pressure (PASP) with transthoracic echocardiography (TTE) in 106 HIV-infected individuals. Participants with a PASP ≥ 30 mmHg (n = 65) underwent right heart catheterization (RHC) to definitively diagnose PAH. We conducted multivariable analysis to identify factors associated with PAH.

**Results:**

Among 106 HIV-infected participants, 80% were male, the median age was 52 years and 77% were on antiretroviral therapy. ET-1 was significantly associated with higher values of PASP [14% per 0.1 pg/mL increase in ET-1, p = 0.05] and PASP ≥ 30 mmHg [PR (prevalence ratio) = 1.24, p = 0.012] on TTE after multivariable adjustment for PAH risk factors. Similarly, among the 65 individuals who underwent RHC, ET-1 was significantly associated with higher values of mean pulmonary artery pressure and PAH (34%, p = 0.003 and PR = 2.43, p = 0.032, respectively) in the multivariable analyses.

**Conclusions:**

Higher levels of ET-1 are independently associated with HIV-associated PAH as hemodynamically assessed by RHC. Our findings suggest that excessive ET-1 production in the setting of HIV infection impairs pulmonary endothelial function and contributes to the development of PAH.

## Introduction

Pulmonary arterial hypertension (PAH) is a rare but serious clinical complication of HIV infection. HIV-associated PAH carries an exceptionally poor prognosis (median survival 0.5 to 3.6 years depending on NYHA functional class) and is an independent risk factor for HIV-related mortality.[1–3] The prevalence of HIV-associated PAH remains approximately 0.5% even in the highly active antiretroviral therapy (ART) era, a rate that has alarmingly not changed compared to that of pre-ART cohorts.[4,5] However, this prevalence does not include asymptomatic individuals—whom we have previously reported to have high pulmonary artery pressures independent of other risk factors for PAH—and thus may underestimate the actual burden of HIV-associated PAH.[6]

Despite recent advances in the pathogenesis of HIV-associated PAH, the mechanisms by which HIV infection causes endothelial dysfunction and subsequent development of PAH remain poorly defined. The HIV proteins Nef and Tat contribute to key processes in PAH including vasoconstriction, cellular proliferation, inflammation, and thrombosis.[7,8] Our group has recently shown that asymmetric dimethylarginine (ADMA), a marker of nitric oxide mediated endothelial dysfunction that accumulates with chronic inflammation, independently predicts HIV-associated PAH.[9,10]

Endothelins are potent vasoconstricting peptides that have proliferative and pro-inflammatory effects, with the isoform ET-1 being a well-established mediator of pulmonary vascular homeostasis.[11] Among other subtypes of PAH, higher levels of endothelin-1 (ET-1) are predictive of severity and mortality, and endothelin receptor antagonists (ERAs) improve clinical and hemodynamic parameters, making them a mainstay of PAH therapy.[12–18] Bosentan, a non-selective ERA, has similarly been shown to improve clinical outcomes in HIV-infected individuals on ART.[19–21] In HIV infection, the HIV envelope glycoprotein gp120 stimulates macrophages and pulmonary arterial endothelial cells to secrete ET-1.[22,23] A recently published study by Feijoo et al. of 23 HIV-infected patients using Doppler transthoracic echocardiography (TTE) demonstrated that plasma levels of ET-1 were higher among individuals with elevated pulmonary artery systolic pressure (PASP) than among uninfected controls and increased with the severity of PASP.[24] While echocardiography is used to screen for PAH, the gold standard for diagnosis of PAH is hemodynamic assessment by right heart catheterization (RHC).[25]

Collectively, these studies support that ET-1 plays a key role in the pathogenesis of HIV-associated PAH. However, the direct relationship between ET-1 and hemodynamically assessed mPAP in HIV-infected individuals has not been studied. The purpose of our study was to evaluate the association between plasma levels of ET-1 and HIV-associated PAH diagnosed by right heart catheterization (RHC). We hypothesized that elevated levels of ET-1 would be associated with higher pulmonary artery pressures, and more specifically, PAH, among HIV-infected individuals and would provide additional support for ET-1 mediated endothelial dysfunction as an important mechanism underlying HIV-associated PAH.

## Methods

### Study Population and Design

We invited HIV-infected participants from the Study of the Consequences of the Protease Inhibitor Era (SCOPE) cohort to undergo Doppler transthoracic echocardiography (TTE) and blood draw for plasma levels of ET-1 and HIV markers using study flyers posted in outpatient clinics. SCOPE is an ongoing, prospective outpatient cohort of greater than 1,500 HIV-infected patients based at San Francisco General Hospital. Documented HIV infection was the only inclusion criterion; we did not use HIV-associated disease characteristics, risk factors for PAH, or symptoms of PAH as selection criteria. There were no exclusion criteria. Non-invasive screening for PAH by TTE (the current standard of practice) was performed on all subjects. Each participant with a PASP ≥ 30 mmHg was offered an invasive RHC to establish a definitive diagnosis of PAH. All study subjects provided written informed consent. The UCSF Committee on Human Research approved this study.

### Participant Characteristics

Each participant completed a comprehensive assessment of demographics (age, gender, race/ethnicity), HIV-related factors, and established risk factors for PAH including the use of stimulants (i.e. cocaine and/or amphetamine/methamphetamine use) both intravenously and via non-parenteral routes. A chart review was also performed to assess duration of HIV infection, nadir CD4+ count (lowest documented value prior to study entry), and ART regimen.

### Blood Measurements

Fasting venous blood was drawn from each subject. Current CD4+ count, HIV RNA level, and lipid panel were measured at the San Francisco General Hospital clinical laboratory. For plasma levels of ET-1, blood samples were immediately centrifuged at 4°C and subsequently stored at -80°C. ET-1 levels were measured using the QuantiGlo ET-1 Chemiluminescent Immunoassay (R&D Systems, Minneapolis, MN).

### Echocardiography

The same experienced sonographer performed all TTEs using a GE Vivid Seven Imaging System (General Electric, Milwaukee, WI). Peak tricuspid regurgitant jet velocity measured by continuous-wave Doppler was used to estimate the pressure gradient between the right ventricle and right atrium via the modified Bernoulli equation.[26] PASP was subsequently calculated by adding this pressure gradient to the right atrial pressure, which was estimated by the respiratory variation in inferior vena cava diameter.[27]

### Right Heart Catheterization

All participants with a PASP ≥ 30 mmHg on TTE were given the opportunity to undergo RHC. The same experienced Interventional Cardiologist (JSM) performed all RHCs following a standard protocol. A 7 French sheath was first inserted into the internal jugular or femoral vein. Next, a balloon-tipped, flow-directed pulmonary artery catheter (Edwards Biosciences, Irvine, CA) was advanced to the right atrium. The pressure transducer was then zeroed at the level of the left atrium. The catheter was ultimately advanced to the pulmonary artery where hemodynamic measurements including mPAP, mean pulmonary capillary wedge pressure (PCWP), cardiac output by Fick’s principle, and pulmonary vascular resistance (PVR) were recorded. Routine measurement of left ventricular end diastolic pressure was not done.

### Definitions and Outcomes

The current accepted definition of pulmonary hypertension is a resting mPAP ≥ 25 mmHg by RHC.[25] PAH (WHO Group 1) is a subgroup of pulmonary hypertension in which the PCWP is ≤ 15 mmHg, the PVR is ≥ 3 Wood units, and other etiologies of pulmonary hypertension including lung disease and chronic thromboembolic disease are absent. Thus, subjects meeting criteria for PAH underwent routine pulmonary function testing, computed tomography of the chest, and ventilation-perfusion imaging to rule out chronic obstructive pulmonary disease, interstitial lung disease, and chronic thromboembolic disease, respectively, to establish a diagnosis of HIV-associated PAH.

We investigated 4 main outcomes: 1) PASP on TTE evaluated as a continuous variable, 2) PASP on TTE evaluated as a dichotomous variable ≥ 30 mmHg, 3) mPAP on RHC evaluated as a continuous variable, and 4) mPAP on RHC evaluated as a dichotomous variable ≥ 25 mmHg with PCWP ≤ 15 mmHg (i.e. PAH).

### Statistical Covariates and Analysis

Candidate covariates of elevated pulmonary arterial pressures included ET-1, demographic characteristics, and established risk factors for PAH. For the univariable analyses, we first built generalized linear regression models with log link function to assess the relationship between ET-1 (independent variable) and both PASP on TTE and mPAP on RHC (dependent variables) and calculate percentage differences in both variables. Next, we fit Poisson regression models using PROC GENMOD to estimate prevalence ratios (PR) and examine the association between each covariate and 1) PASP ≥ 30 mmHg, and 2) mPAP ≥ 25 mmHg and PCWP ≤ 15 mmHg. We applied the model-robust sandwich variance estimator to all analyses because when Poisson regression is applied to a binary outcome, the standard error is overestimated, leading to wider confidence intervals.[28–29] For the multivariable models, we applied a penalized regression method (LASSO regression) to select candidate covariates associated with continuous outcome variables, while forcing in demographic covariates.[30] This method allowed for the selection of relevant covariates and the estimation of their regression coefficients in final models. We fit the multivariable Poisson regression models with the covariates selected by LASSO regression. Analyses were performed using the SAS system, version 9.3 (SAS Institute, Inc., Cary, NC) and R package glmnet (version 3.1.2).

## Results

### Participant Characteristics

We measured levels of ET-1 in 106 HIV-infected participants (Table 1). Overall, the median (interquartile range [IQR]) age was 52 years (IQR 44–57), 80% were men, 9% had current intravenous drug use (IVDU), and 20% were currently using stimulant drugs non-parenterally. The median duration of HIV infection was 16 years (IQR 10–22), 77% were on ART, the median current CD4+ count was 591 cells/μL (IQR 366–757), and 69% had an undetectable viral load.

**Table 1:** Baseline Characteristics of HIV-Infected Subjects.

Parameter	All Subjects (N = 106)	RHC (N = 65)	Non-RHC (N = 22)	p-value
Age (yr)	52 (44–57)	52 (46–57)	48 (42–57)	0.27
Male	85 (80%)	54 (83%)	14 (64%)	0.03
Race				0.61
Caucasian	53 (50%)	33 (51%)	8 (36%)	
African American	34 (32%)	20 (31%)	10 (45%)	
Latino	11 (10%)	7 (11%)	2 (9%)	
Other	8 (8%)	5 (7%)	2 (9%)	
Stimulant IVDU (ever)	43 (41%)	33 (51%)	6 (27%)	0.06
Stimulant IVDU (current)	10 (9%)	8 (12%)	2 (9%)	0.68
Stimulant Non-Parenteral Use (ever)	63 (59%)	41 (63%)	11 (50%)	0.28
Stimulant Non-Parenteral Use (current)	21 (20%)	17 (26%)	2 (9%)	0.09
Duration of HIV Infection (yr)	16 (10–22)	17 (12–22)	16 (12–21)	0.87
ART Use (ever)	88 (83%)	59 (91%)	19 (86%)	0.56
ART Use (current)	82 (77%)	55 (85%)	18 (82%)	0.76
ART Duration (yr)	4.5 (1.0–9.4)	5.8 (1.8–9.6)	5.9 (1.4–10.1)	0.80
Current CD4+ (cells/μL)	591 (366–757)	637 (376–768)	430 (231–672)	0.04
Nadir CD4+ (cells/μL)	184 (40–268)	133 (38–250)	172 (43–264)	0.74
HIV RNA (Viral Load)				0.81
<75 (copies/mL)	73 (69%)	49 (75%)	15 (68%)	
75–1999 (copies/mL)	15 (14%)	7 (11%)	2 (9%)	
2000–9999 (copies/mL)	6 (6%)	2 (3%)	1 (5%)	
>10000 (copies/mL)	12 (11%)	7 (11%)	4 (18%)	

ART, antiretroviral therapy; IVDU, intravenous drug use; RHC, right heart catheterization.

Data are presented as median (interquartile range) or numbers (percent).

### Relationship between ET-1 and PASP on TTE

The overall median level of ET-1 was 1.78 pg/ml (IQR 1.38–2.18). Among the 19 subjects with a PASP < 30 mmHg and 87 subjects with a PASP ≥ 30 mmHg, the median levels of ET-1 were 1.38 pg/ml (IQR 1.19–1.73) and 1.89 pg/ml (IQR 1.48–2.32), respectively. Elevated levels of ET-1 predicted higher values of PASP (14% per 0.1 pg/ml increase in ET-1, p = 0.05, Table 2A) and increased risk of having PASP ≥ 30 mmHg (PR = 1.24 per 0.1 pg/ml increase in ET-1, p = 0.012, Table 2B) in the univariable analysis. These associations remained statistically significant after multivariable adjustment (PASP: 18%, p = 0.023, Table 2A; PASP ≥ 30 mmHg: PR = 1.27, p = 0.013, Table 2B). Additionally, stimulant IVDU and female gender were independently associated with increased PASP (Table 2A), while stimulant IVDU was also independently associated with PASP ≥ 30 mmHg (Table 2B).

**Table 2:** Factors Associated with PASP on TTE in HIV-Infected Subjects.

**A. PASP**		
	**Univariable**	**Multivariable**
**Parameter**	% Estimate (95% CI), p-value	% Estimate (95% CI), p-value
ET-1 (per 0.1 pg/ml increase)	14% (0%, 31%), **p = 0.050**	18% (2%, 36%), **p = 0.023**
Age (per decade)	-8% (-16%, 1%), p = 0.090	-8% (-16%, 0%), p = 0.064
Male vs. Female	-18% (-33%, -1%), **p = 0.037**	-18% (-32%, -1%), **p = 0.035**
African-American vs. Caucasian	3% (-11%, 18%), p = 0.74	-4% (-17%, 10%), p = 0.51
Latino vs. Caucasian	16% (-16%, 62%), p = 0.37	22% (-9%, 64%), p = 0.18
Other vs. Caucasian	-8% (-21%, 7%), p = 0.28	-3% (-18%, 15%), p = 0.71
Stimulant IVDU (ever vs. never)	21% (5%, 40%), **p = 0.009**	16% (0%, 34%), **p = 0.046**
**B. PASP ≥ 30 mmHg**		
	**Univariable**	**Multivariable**
**Parameter**	PR (95% CI), p-value	PR (95% CI), p-value
ET-1 (per 0.1 pg/ml increase)	1.24 (1.05, 1.48), **p = 0.012**	1.27 (1.05, 1.52), **p = 0.013**
Age (per decade)	1.00 (0.97, 1.04), p = 0.804	1.00 (0.97, 1.04), p = 0.989
Male vs. Female	0.84 (0.73, 0.97), **p = 0.016**	0.87 (0.73, 1.05), p-0.145
African-American vs. Caucasian	1.18 (0.98, 1.41), p = 0.081	1.13 (0.92, 1.39), p = 0.240
Latino vs. Caucasian	1.06 (0.77, 1.45), p = 0.727	1.08 (0.77, 1.51), p = 0.661
Other vs. Caucasian	1.13 (0.84, 1.53), p = 0.42	1.20 (0.89, 1.61), p = 0.237
Stimulant IVDU (current vs. not current)	1.17 (0.99, 1.38), **p<0.001**	1.28 (1.09, 1.51), **p = 0.002**

CI, confidence interval; ET-1, endothelin-1; IVDU, intravenous drug use; PASP, pulmonary artery systolic pressure; PR, prevalence ratio; TTE, transthoracic echocardiography.

Analysis includes all 106 HIV-infected subjects. PASP is log-transformed; results are back-transformed to calculate estimated percentage differences in PASP attributable to each covariate.

### Relationship between ET-1 and PAH on RHC

Of the 87 participants with a PASP ≥ 30 mmHg on TTE, 65 (75%) consented to undergo an elective RHC. This RHC subgroup had a significantly higher percentage of men and current CD4+ counts but similar nadir CD4+ counts and HIV RNA levels compared to the non-RHC cohort (Table 1). Among the RHC subgroup, 16 (25%) subjects fulfilled the hemodynamic criteria described above to establish a diagnosis of true PAH (Table 3). Of these 16 individuals with PAH, 4 (25%) were female and all were on ART and asymptomatic. Among the 47 subjects with a mPAP < 25 mmHg, the median level of ET-1 was 1.81 pg/ml (IQR 1.46–2.20). The median level of ET-1 was 2.22 pg/ml (IQR 1.79–2.99) among the 16 subjects with PAH. Higher levels of ET-1 predicted increased values of mPAP (28% per 0.1 pg/ml increase in ET-1, p = 0.06, Table 4A) and higher risk of PAH (PR = 2.35 per 0.1 pg/ml increase in ET-1, p = 0.033, Table 4B) in the univariable analysis. These associations remained statistically significant in the multivariable analysis (mPAP: 34%, p = 0.003, Table 4A; PAH: PR = 2.39, p = 0.028, Table 4B). Among subjects with PAH, current and nadir CD4+ counts showed little association with levels of ET-1 (data not shown). Older age was also independently associated with increased mPAP (Table 4A).

**Table 3:** Hemodynamic Assessment by RHC of HIV-Infected Subjects with PAH.

Participant	mPAP (mmHg)	PCWP (mmHg)	CO (L/min)	PVR (WU)
1	32	5	3.7	7.3
2	25	8	3.6	4.7
3	42	9	5.3	6.2
4	27	12	4.0	3.8
5	46	10	3.9	9.2
6	34	10	4.4	5.5
7	48	5	3.5	12.3
8	30	8	6.4	3.4
9	54	8	5.0	9.2
10	26	12	4.0	3.5
11	25	14	3.6	3.1
12	51	6	6.8	6.6
13	29	10	5.0	3.8
14	32	7	3.1	8.1
15	60	7	4.8	11
16	68	8	3.8	15.8

CO, cardiac output (measured by Fick’s principle); mPAP, mean pulmonary arterial pressure; PAH, pulmonary arterial hypertension; PCWP, pulmonary capillary wedge pressure; PVR, pulmonary vascular resistance; RHC, right heart catheterization; WU, Wood units.

**Table 4:** Factors Associated with mPAP on RHC in HIV-Infected Subjects.

**A. mPAP**		
	**Univariable**	**Multivariable**
**Parameter**	% Estimate (95% CI), p-value	% Estimate (95% CI), p-value
ET-1 (per 0.1 pg/ml increase)	28% (-1%, 67%), p = 0.060	34% (11%, 63%), **p = 0.003**
Age (per decade)	-16% (-28%, -2%), **p = 0.031**	-17% (-29%, -4%), **p = 0.015**
Male vs. Female	1% (-15%, 19%), p = 0.946	3% (-15%, 26%), p = 0.733
African-American vs. Caucasian	-10% (-28%, 13%), p = 0.382	-1% (-18%, 20%), p = 0.918
Latino vs. Caucasian	2% (-33%, 55%), p = 0.926	12% (-25%, 68%), p = 0.567
Other vs. Caucasian	-28% (-46%, -5%), **p = 0.023**	-10% (-26%, 10%), p = 0.326
Stimulant IVDU (ever vs. never)	25% (0%, 56%), **p = 0.047**	16% (-4%, 40%), p = 0.137
**B. PAH (mPAP ≥ 25 mmHg)**		
	**Univariable**	**Multivariable**
**Parameter**	PR (95% CI), p-value	PR (95% CI), p-value
ET-1 (per 0.1 pg/ml increase)	2.35 (1.07, 5.13), **p = 0.033**	2.39 (1.10, 5.20), **p = 0.028**
Age (per decade)	0.77 (0.49, 1.20), p = 0.246	0.69 (0.41, 1.14), p = 0.145
Male vs. Female	0.88 (0.30, 2.59), p = 0.820	0.96 (0.34, 2.70), p = 0.940
African American vs. Caucasian	0.92 (0.36, 2.35), p = 0.856	1.17 (0.44, 3.11), p = 0.754
Latino/Other vs. Caucasian	0.61 (0.15, 2.43), p = 0.485	1.05 (0.24, 4.56), p = 0.950

CI, confidence interval; ET-1, endothelin-1; IVDU, intravenous drug use; mPAP, mean pulmonary artery pressure; PAH, pulmonary arterial hypertension; PR, prevalence ratio; RHC, right heart catheterization.

Analysis includes the subgroup of 65 HIV-infected subjects who underwent RHC. mPAP is log-transformed; results are back-transformed to calculate estimated percentage differences in mPAP attributable to each covariate.

## Discussion

**Fig 1:**
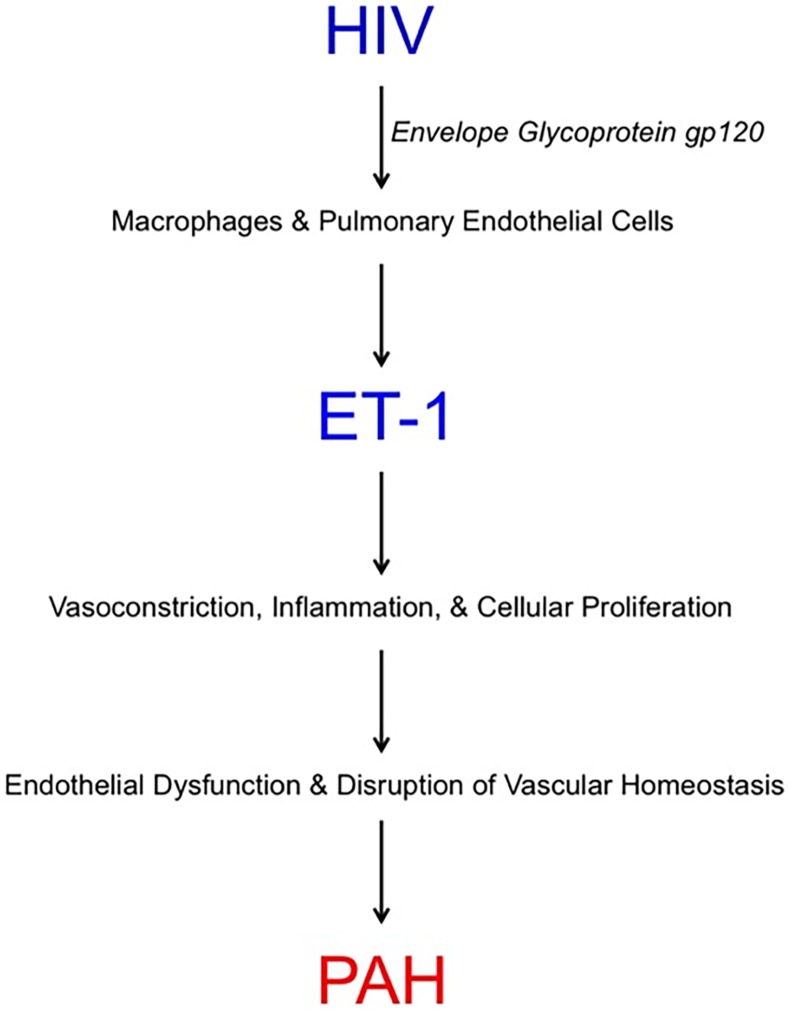
Proposed Mechanism by which ET-1 Contributes to the Development of HIV-Associated PAH. ET-1, endothelin-1; PAH, pulmonary arterial hypertension.

The pathogenesis of PAH in the setting of HIV infection remains poorly understood despite new insights into the mechanistic roles of ADMA and the HIV proteins Nef and Tat.[7–9] A recent study by Feijoo et al. reported that plasma levels of ET-1 increased as the severity of HIV-associated PAH assessed non-invasively on TTE progressed.[24] In the present study, we observed a similar relationship between elevated levels of ET-1 and higher pulmonary artery pressures estimated by TTE. However, we expand upon these findings by demonstrating that, among HIV-infected individuals, elevated levels of ET-1 are independently associated with hemodynamically assessed PAH by invasive RHC (the diagnostic gold standard). This relationship has not been previously described to our knowledge and importantly provides additional evidence that ET-1 contributes to the pathogenesis of HIV-associated PAH (Fig 1).

Our study and that of Feijoo et al. had several key differences. First, they used a cutoff of PASP ≥ 35 mmHg on TTE to diagnose PAH. While echocardiography is commonly used to screen for elevated PASP, a formal diagnosis of PAH requires direct hemodynamic assessment via RHC. In our study, of the 42 individuals who had a PASP ≥ 35 mmHg on TTE and underwent RHC, only 15 had true PAH. Using echocardiography alone to identify PAH as was done in the study by Feijoo et al. would have misclassified 64% of these individuals, highlighting the limitations of echocardiography to establish a diagnosis of PAH. We employed a cutoff of ≥ 30 mmHg to screen subjects for PAH because 1) our group recently found that using the lower threshold resulted in less missed cases of PAH among HIV-infected individuals, and 2) a PASP ≥ 30 mmHg on TTE has been shown to predict mortality in certain patient populations who are at risk for PAH.[31,32] Next, Feijoo et al. reported an overall median level of ET-1 of 1.16 pg/ml (IQR 0.86–2.37) among 23 HIV patients with elevated PASP on TTE. In comparison, the level of ET-1 among 16 subjects diagnosed with HIV-associated PAH on RHC in our study was 2.22 pg/ml (IQR 1.79–2.99), which is significantly higher and likely reflects a greater disease burden. For example, 38% of our PAH cohort had severe PAH as compared to 17% of their cohort. Finally, Feijoo et al. did not report a statistically significant difference in levels of ET-1 between HIV-infected patients with and without elevated PASP on TTE. In contrast, we observed an independent relationship between ET-1 and PASP as both a continuous and dichotomous variable among our HIV cohort. This difference is likely due to a smaller sample size (n = 68 vs. n = 106) and thus less power to detect an association in the Feijoo et al. study.[24]

HIV-associated PAH carries an exceedingly poor survival rate and independently predicts HIV-specific mortality, rendering it one of the most devastating non-AIDS complications of HIV infection.[1–3] In fact, the survival rate is worse than those in other forms of PAH according to some reports.[1,33] The reported prevalence rate of 0.5% reflects symptomatic patients carrying a clinical diagnosis of HIV-associated PAH but fails to capture the asymptomatic PAH population.[4,5] In our study, 15% of an asymptomatic HIV cohort was found to have subclinical PAH. Thus, earlier recognition of HIV-associated PAH including intervention before development of symptoms may play a role in improvement in clinical outcomes. Our study showed that higher levels of ET-1 are independently associated with HIV-associated PAH in an asymptomatic and previously undiagnosed cohort. The levels of ET-1 in our HIV-associated PAH cohort [median 2.22 pg/ml (IQR 1.79–2.99)] are substantially higher than those observed in healthy individuals; for example, Feijoo et al. reported a median ET-1 level of 0.71 pg/ml (IQR 0.54–0.94) among 11 controls.[22,34,35] Prior studies of other forms of PAH demonstrated that elevated levels of ET-1 are correlated with worse hemodynamic features and mortality; the ET-1 levels in those series were higher than in our study, reflecting more cases of severe PAH.[13,15–17] Taken together, these findings suggest that ET-1 may be considered as both a screening biomarker and prognostic tool for HIV-associated PAH, though additional longitudinal studies with outcome data would be required for validation.

As a principal mediator of pulmonary vascular homeostasis, ET-1 has long been a chief therapeutic target in PAH in the form of ERAs. Two distinct receptor subtypes mediate the bioactivity of ET-1: 1) ET-A receptors on vascular smooth muscle cells, and 2) ET-B receptors on both endothelial and vascular smooth muscle cells. Stimulation of the vascular smooth muscle cell ET-A and ET-B receptors causes vasoconstriction whereas binding of ET-1 to endothelial ET-B receptors triggers the release of the potent vasodilators NO and prostacyclin.[11] Therefore, selective blockade of ET-A receptors may theoretically lead to greater overall vasodilation compared to non-selectively blocking both ET-A and ET-B receptors. Bosentan, a non-selective ERA, is the only ERA that has been studied in clinical trials focused on HIV-associated PAH. It has been shown to improve both clinical and hemodynamic parameters in patients with advanced disease on ART.[19–21] The ET-A selective ERA Ambrisentan was extensively studied in 3 sequential trials of PAH (overall N = 393, 11 of whom had HIV-associated PAH) and was found to similarly improve pulmonary hemodynamics and delay clinical progression.[36–38] Head-to-head studies of non-selective versus selective blockade of ET-1 receptors are needed to establish the first-line ERA in HIV-associated PAH. Future investigation is also required to determine if ERAs delay the development of PAH or the onset of symptoms in asymptomatic PAH in this population.

Though ET-1 was the only candidate covariate to demonstrate an independent association with all outcomes including PAH, we detected significant associations for both female gender and stimulant IVDU with certain measures of pulmonary pressure. PAH registries report an overall gender influence with a female to male ratio of nearly 4:1 for all etiologies of PAH. However, HIV-associated PAH specifically exhibits a male predominance given that the overwhelming majority of HIV-infected individuals are men.[39,40] We observed that female gender was significantly associated with PASP on TTE, but notably not with PAH by RHC; overall, our findings are consistent with the registry data. Lastly, the significant associations we found between stimulant IVDU and various measures of pulmonary pressure were expected given that it is a known risk factor for PAH.

The present study has key limitations. First, the study’s cross-sectional design prevents the determination of causality. Though a mechanistic link exists between HIV infection, ET-1, and PAH (HIV gp120 stimulates macrophages and pulmonary arterial endothelial cells to secrete ET-1), a larger longitudinal study showing that elevated levels of ET-1 predict the development of PAH in HIV-infected patients and are higher than those in uninfected controls would be required to establish a causal relationship and support ET-1 as a potential biomarker of HIV-associated PAH, respectively.[22,23] Nonetheless, our observations are consistent with the results of the recent study by Feijoo et al. and previous observations in cross-sectional studies of other subtypes of PAH, and thus reinforces that ET-1 is one of the mechanisms by which HIV-associated PAH develops.[13,15–17,24] Second, we did not directly measure pulmonary endothelial ET-1 levels. However, ET-1 expression is highest in the pulmonary vasculature and PAH patients have higher circulating plasma levels of ET-1 as well as increased ET-1 levels exiting the lung than entering the lung.[11,41] These observations suggest that the elevated plasma levels of ET-1 we observed in the HIV-associated PAH cohort likely reflects impaired pulmonary endothelium. Third, the impact of ET-1 compared to other predictors on HIV-associated PAH is unclear. Serial measurement of Nef, Tat, ADMA, and ET-1 levels over time in an HIV population would help shed light on the relative contributions of each of these mechanistic factors. Lastly, the inherent inaccuracy of TTE in measuring pulmonary artery pressure is a fundamental limitation. Doppler echocardiography, when compared to the gold standard of RHC, inaccurately estimates PASP in approximately 50% of the cases in general PAH cohorts (defined as > ±10 mmHg difference between TTE and RHC).[42,43] Similarly, our group recently reported that among HIV-infected patients, TTE inaccurately estimated PASP in 20% of the cases and that 29% of diagnoses of HIV-associated PAH were missed using a screening threshold of PASP ≥ 30 mmHg on TTE.[31] Collectively, these findings suggest that we may have failed to detect cases of PAH in this study among individuals with a PASP < 30 mmHg on TTE and highlights the need for a more sensitive tool than echocardiography to screen for HIV-associated PAH.

In summary, elevated plasma levels of ET-1 are independently associated with PAH diagnosed by RHC in the setting of HIV infection. Our study is the first to our knowledge to link ET-1 to hemodynamically assessed HIV-associated PAH, thus extending upon the existing echocardiographic literature. This finding supports our hypothesis that ET-1 disrupts pulmonary endothelial function and plays a mechanistic role in the development of HIV-associated PAH. Future studies in HIV-infected patients are required to determine sensitive ET-1 thresholds for earlier recognition of PAH and to assess if higher levels of ET-1 predict functional or clinical outcomes. Additionally, the possibility of using ET-1 to predict success of endothelin receptor antagonism in this population warrants further investigation. HIV-infected individuals (Pulido T NEJM 2013). Larger investigations targeted at use of ET-1 levels and lowering ET-1 with ERA in HIV-PAH will be needed to establish the efficacy and safety of the different ERA in HIV. In addition, the benefit of lowering ET-1 among asymptomatic HIV-infected individuals with the purpose of delaying development of PAH or delaying symptom onset remains unknown.
